# Imaging of pediatric bone and growth disorders: Of diagnostic workhorses and new horizons

**DOI:** 10.1007/s10354-021-00815-z

**Published:** 2021-02-11

**Authors:** Sarah N. Mehany, Janina M. Patsch

**Affiliations:** 1 Department of Biomedical Imaging and Image-Guided Therapy, Medical University of Vienna, Vienna, Austria; 2 Vienna Bone and Growth Center, Vienna General Hospital and Medical University of Vienna, Vienna, Austria

**Keywords:** Osteogenesis imperfecta, X-linked hypophosphatemic rickets, Achondroplasia, X-rays, Magnetic resonance imaging

## Abstract

Children and adolescents with bone and growth disorders require interdisciplinary care from various specialists including pediatric radiologists with a focus on musculoskeletal disorders. This article covers routine topics, differential diagnoses, and selected research imaging in children with osteogenesis imperfecta (OI), X‑linked hypophosphatemic rickets (XLH), achondroplasia, and other bone and growth disorders from the standpoint of a tertiary referral center.

## Abstract (de)

Kinder und Jugendliche mit seltenen Knochenerkrankungen und Wachstumsstörungen bedürfen einer interdisziplinären Expertenbetreuung. In diesem Zusammenhang stellt die enge Zusammenarbeit mit einer Kinderradiologie mit muskuloskeletalem Schwerpunkt eine essenzielle Säule der medizinischen Versorgung dar. Der vorliegende Übersichtsartikel umfasst Aspekte der radiologisch-diagnostischen Grundversorgung sowie ausgewählte Themen zur bildgebenden Forschung bei Kindern mit Osteogenesis imperfecta (OI), Phosphatdiabetes (XLH), Achondroplasie und anderen seltenen Knochenerkrankungen aus dem Blickwinkel eines akademischen Tertiärzentrums.

## Introduction

Pediatric bone and growth disorders require interdisciplinary medical care with a central role for diagnostic imaging. Basic principles of pediatric imaging apply to all children, but in particular to patients with chronic and complex conditions. Rigorous radiation protection according to the ALARA (as low as reasonably achievable) principle, child-friendly protocols in cross-sectional imaging, and critical approach to intravenous use of contrast material (gadolinium-based agents in magnetic resonance imaging [MRI]; iodinated agents in computed tomography [CT]) are essential (www.imagegently.org).

Of importance, sedation should be avoided whenever possible through age-adequate handling and trained staff including technician and child-life specialists. If sedation is needed (e.g., cross-sectional imaging in very young children or children with special needs) it should be performed by anesthesiologists with dedicated pediatric protocols and experience in pediatric medicine [1]. In neonates, feed-and-wrap techniques can be used to achieve diagnostic image quality [2].

But the role of a pediatric radiologist at a tertiary referral center for pediatric bone and growth disorders reaches beyond. Especially in children with rare diseases or complex conditions, involvement of imaging experts is of pivotal importance. With images illustrating case presentations and discussions, the pediatric radiologist acts as central player in interdisciplinary boards and therein supports clinical decision making and problem solving.

## Routine imaging: Methods and their use

### Radiographs

**Fig. 1:**
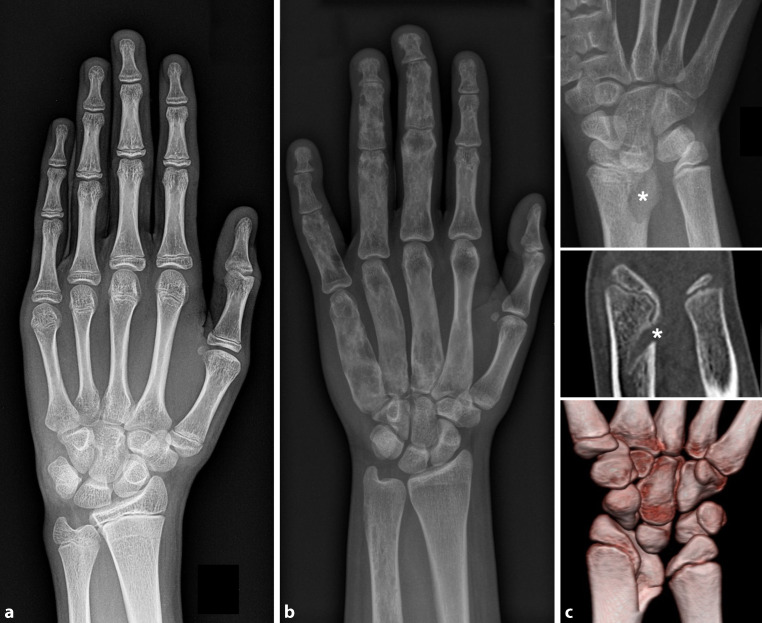
Conventional hand radiographs.** a** Healthy 15-year-old girl. **b** Polyostotic fibrous dysplasia in McCune–Albright syndrome. **c** Madelung deformity in a girl with SHOX-deficiency (*top*: radiographs; *middle* and *bottom*: computed tomography with 3D reconstruction). *Asterisks* highlight the bony deformities of the distal radius (with ulnar and volar tilting, decreased carpal angle) and the pathologic thickening of the short radiolunate ligament (Vicker’s ligament). There is secondary triangularization of the lunate

Conventional radiographs are a basic diagnostic tool in pediatric bone imaging (Fig. 1). In children with bone and growth disorders, typical indications include the assessment of bone age (e.g., in endocrine diseases), the detection and characterization of physeal abnormalities (e.g., signs of rickets with widening, cupping, and fraying in X‑linked hypophosphatemic rickets [XLH]), and leg alignment (e.g., in XLH). Further, conventional radiographs set the basis for the detection, characterization, and monitoring of osseous lesions (e.g., in McCune–Albright syndrome, a disorder with multiple endocrinopathies, extensive polyostotic fibrous dysplasia, and café-au-lait macules [3]), abnormal soft tissue calcifications (e.g., fibrodysplasia ossificans progressiva [FOP], a disorder that leads to extensive and progressive extra-skeletal ossifications [4]), fractures (e.g., osteogenesis imperfecta), and deformities (e.g., Madelung deformity in SHOX-mutations). Skeletal surveys complement, support, and consolidate clinical assessment in cases of suspected skeletal dysplasias [5]. Moreover, conventional radiographs complete surgical planning, and aid post-surgical monitoring.

### Routine ultrasound

Ultrasound is the workhorse of pediatric imaging. With adequate levels of operator expertise (e.g., medical doctors or sonographers with specific training in pediatric ultrasound), excellent accuracy and reproducibility can be achieved. In children with bone and growth disorders, endocrine, nephrologic, and orthopedic requests are most common (e.g., to rule out, diagnose and/or quantify nephrocalcinosis or renal stones in calciuric or phosphaturic conditions; locate and measure gonads; search for tumors in predisposition syndromes or suspected ectopic hormone production; joint effusions or fractures). In children with chronic diseases, vascular ultrasound aids to define optimal puncture site/vessel for continuous venous access and to diagnose vascular complications. In newborns, hip screening programs are different from country to country. Nevertheless, using either the Graf or the Harke method, development dysplasia of the hip (DDH) can be easily diagnosed, triaged for therapy, and monitored [6]. In newborns with genetic/syndromic conditions “teratologic” luxations are known and complicated to treat. On the other hand, hip scans of newborns with skeletal dysplasias should only be performed by expert operators because both acquisition and interpretation are difficult (if even possible by state-of-the-art methods).

### Dual-energy X-ray absorptiometry

Dual-energy X‑ray absorptiometry (DXA) is the gold standard technique for assessing areal bone mineral density in both children and adults. The radiation dose is very low, corresponding to about a day of physiologic background radiation in most European countries [7].

**Fig. 2:**
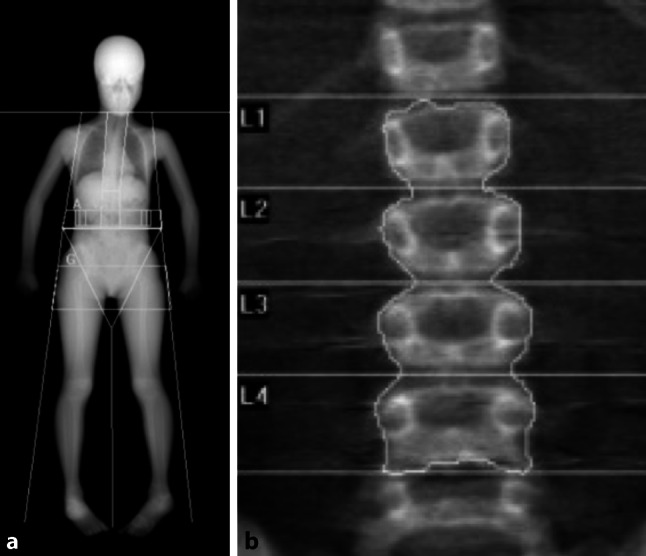
Recommended measurement sites in pediatric dual-energy X‑ray absorptiometry (DXA). **a** Total body less head (TBLH), **b** lumbar spine

While in adults the lumbar spine and the hip are defined as standard measurement sites, the recommended pediatric sites are not identical. In children, the hip should not be measured. Instead a whole body scan is acquired and the head excluded (total body less head [TBLH]; Fig. 2). As opposed to adults, there are no T‑scores in children. Z‑scores are used, and as a consequence the terms osteopenia and osteoporosis are not applicable to pediatric DXA interpretation. The pathologic threshold lies at Z‑score ≤ −2 and the correct term to be used in cases with abnormal density is “low bone mass” [8, 9]. In the presence of clinical fragility fractures, a child can be formally diagnosed with “juvenile or secondary osteoporosis”, but the definition does not rely on low bone mineral density (BMD) alone.

In addition, pediatric Z‑scores should be normalized for subject height. This is relevant because—due to the nature of the method—DXA only determines areal BMD which is overestimated in large bone and underestimated in small bones. Thus, in children with bone and growth disorders two systematic errors coincide because patients are often too short for their age. Typically, adjustment for height is automatically performed after the scan by the inbuilt software of the DXA machine. Physicians providing pediatric DXA reports must therefore be familiar with the concept of pediatric adjustments (e.g., height, weight). Adjusted Z‑scores can also be obtained from a web-based electronic calculator published by the Childrens’ Hospital of Philadelphia (https://zscore.research.chop.edu/bmdCalculator.php) [10]. Moreover, both DXA technician and physician have to ensure that metal hardware is properly excluded from the density analysis (e.g., rods in children with osteogenesis imperfecta).

### Magnetic resonance imaging and computed tomography

In children with bone and growth disorders, cross-sectional imaging methods typically come into play when osseous lesions including bone marrow edema or spine fractures are suspected. In these settings, cross-sectional imaging tools are used due to their superiority to other techniques (e.g., imaging of the craniocervical junction in children with achondroplasia or other skeletal dysplasias affecting this region).

### Magnetic resonance imaging (MRI)

MRI is an imaging method which provides excellent depiction and contrast of soft tissue. The technique uses a static magnetic field and electromagnetic gradients to induce nuclear spinning and relaxation phenomena in the human body. Signals are received by dedicated coils and transformed into a diagnostic MR image. Different weighing and sequences compose a multiparametric exam. MRI is free of ionizing radiation and is therefore considered preferential to computed tomography in pediatric imaging. Importantly, MR-specific safety issues have to be considered: Ferromagnetic objects must be strictly avoided. Heating can occur. Medical devices (e.g., such as stents, implants, dental braces) require rigorous and time-consuming safety work-up (www.mrisafety.com).

Traditionally, musculoskeletal conditions and neurological diseases have been classical fields of indication (e.g., imaging of the spinal cord, the vertebral column including ligaments and discs; brain imaging and peripheral nerve imaging). Due to the morphology of the growing skeleton, MRI often allows a more in-depth look: Permanent and transient cartilaginous structures such as articular cartilage, growth plates, epiphyses, and apophyses can be directly visualized [11]. Bone marrow properties and changes including age-dependent conversion from red to yellow marrow or its reconversion in chronic hematopoetic conditions can be well assessed on MR images [12]. Torsional analysis of the lower limbs have shifted from CT to (hip–knee–ankle) MRI [13]. Whole-body MRI can be used to reveal the extent of disease in systemic or multifocal diseases [14].

### Computed tomography (CT)

The idea of needing a CT scan in a child, often evokes significant stress in parents and even pediatricians. In this context, it is important to understand that modern CT machines and protocols optimized for children deliver less radiation than widely assumed. Besides that, for certain indications and clinical settings pediatric CT remains preferential over pediatric MRI, e.g., assessment of cortical bone lesions, children with metal rods (e.g., after surgery for scoliosis, limb-lengthening, or osteogenesis imperfecta), or children with pacemakers without MR compatibility. Overall, scan time of CT is much faster than scan time of a typical MRI.

## Examples for common diagnostic scenarios at a tertiary referral center

### Pediatric and juvenile spine fractures

For the differential diagnosis of pediatric or juvenile spine fractures specific conditions and diseases have to be considered, e.g., post-traumatic fractures versus low trauma fractures or congenital deformities. Primary or secondary bone lesions can lead to compromised local bone strength and consecutive vertebral collapse (e.g., eosinophilic granuloma/Langerhans’ histiocytosis, metastatic disease, hemato-oncologic disease with diffuse infiltration of bone marrow). Chronic non-infectious osteomyelitis (CNO)/chronic recurrent multifocal osteomyelitis (CRMO) is visible as focal bone marrow edema (on MR) with or without fractures.

**Fig. 3:**
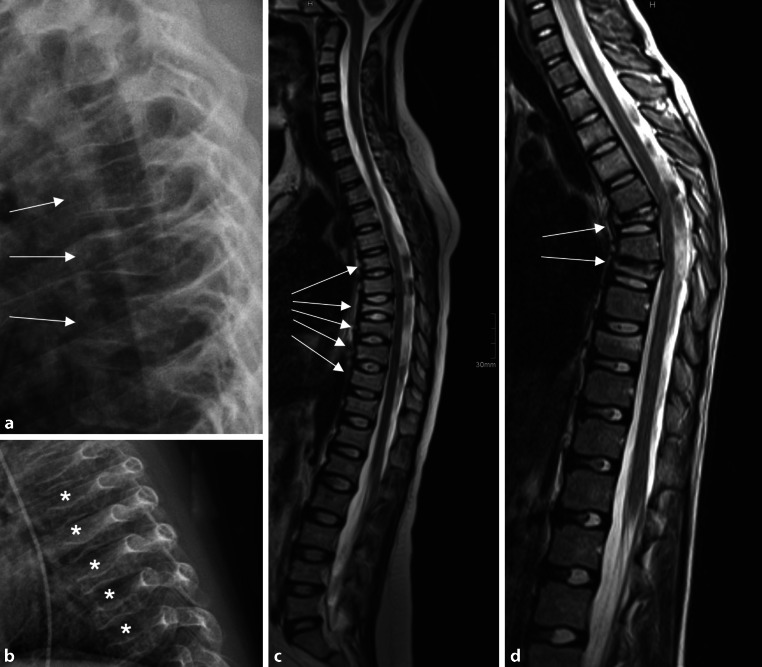
Pediatric and adolescent spine fractures. **a** Osteogenesis imperfecta. *Arrows *fractured vertebrae (anterior wedging). **b** Secondary osteoporosis (after heart transplantation). *Asterisks* indicate the preserved and relatively wide disc spaces, a typical feature of pediatric spinal insufficiency fractures. **c** Idiopathic osteoporosis. Vertebral fractures (*arrows*) on magnetic resonance imaging (MRI). **d** Chronic non-infectious osteomyelitis (CNO)/chronic recurrent multifocal osteomyelitis (CRMO). Severe fractures (*arrows*) leading to thoracic gibbus deformity

Importantly, pediatric spine fractures look different on imaging than classic osteoporotic or age-related fractures. Intervertebral discs are high, their water content is maintained. In fact, increased disc height—relative to reduced vertebral body height—can be a useful reference in pediatric spine fractures (Fig. 3). It should also be kept in mind that skeletal growth provides unique potential to normalize low bone mass and to reshape fractured vertebral bodies.

In eosinophilic granuloma, flattened vertebral bodies (“vertebra plana”) can be found as a characteristic imaging sign. This should not be confused with platyspondyly (i.e., diffuse flattening of all vertebrae).

### Osteogenesis imperfecta

**Fig. 4:**
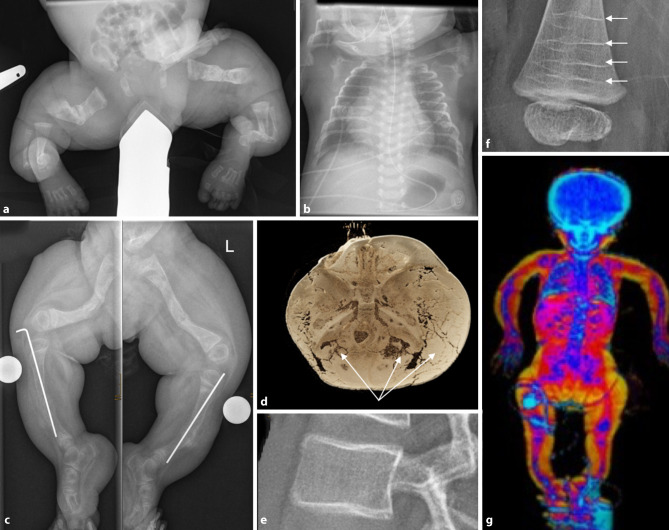
Osteogenesis imperfecta. **a** Multiple congenital fractures, **b** slender, gracile ribs, **c** orthopedic pin stabilization of the tibia. **d** Three-dimensional reconstruction of skull base (by computed tomography). Visibility of multiple wormian bones (*arrows*). **e** Teenager with mild form of osteogenesis imperfecta (OI). Cortical framing after bisphosphonate therapy, **f** (bisphosphonate-induced) metaphyseal Zebra lines (*arrows*), **g** dual-energy X‑ray absorptiometry (DXA) whole-body image (short stature, extremity bowing, macrocephaly). Please note this is the same child as in **a**, **b** (as a newborn), now 3 years old

Children and adolescents with various forms of osteogenesis imperfecta (OI) are a major patient cohort of the Vienna Bone and Growth Center. Often, fetal imaging with significant shortening and bowing of extremities starts the diagnostic cascade and planning of delivery at a tertiary referral center. In the postnatal phase a skeletal survey is mandatory to understand the extent of pre-existing fractures (Fig. 4). The occurrence of new fractures is typically suspected on a clinical basis and confirmed by radiographs. The differentiation of OI-related fractures and non-accidental injury (=child abuse) can be difficult, especially in young children with undiagnosed OI [15, 16].

The effects of cyclic administration of bisphosphonates are also documented by an imaging epi-phenomenon referred to as the “zebra line sign” ([17]; Fig. 4f). Hypertrophic callus formation can be observed in OI type V [18]. At the calvaria multiple wormian bones can be found (Fig. 4d), but skull involvement varies.

Furthermore in OI, repeated radiographs are needed to plan and follow-up extremity surgery with intramedullary pins and to assess fracture status at the spine. The OI group of the University of Cologne has published and established a scoring method to facilitate reading, reporting, and comparability of lateral spine radiographs in OI [19].

DXA is a helpful tool to monitor treatment effects in OI but metal hardware needs to be meticulously excluded from the quantitative analysis (Fig. 4g). At certain centers, lateral DXA scans have successfully replaced lateral spine x‑rays in children with OI.

### X-linked hypophosphatemic rickets (XLH)

**Fig. 5:**
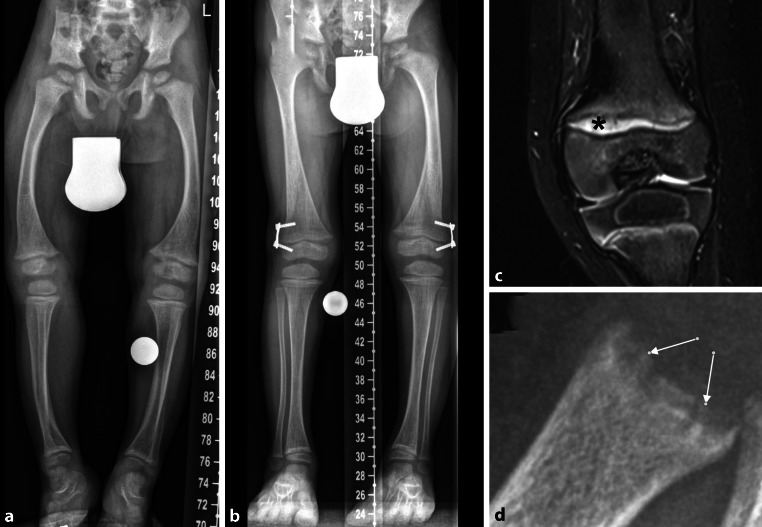
X‑linked hypophosphatemic rickets. **a** Leg bowing with **b** significant improvement after bilateral hemiepiphysiodesis. **c** Physeal widening in MRI (*asterisk* placed on abnormal physis). **d** Rickets on conventional radiographs (physeal cupping and fraying; *arrows*)

Children with XLH present with radiographic signs of rickets which are similar to nutritional rickets with physeal widening, cupping, and fraying [20]. Consequently, Thatcher scoring (RSS: rickets severity score) which has been established in nutritional rickets is also applicable to XLH [21]. More recently, some XLH centers have started to use MRI of the knee as a diagnostic tool and as a monitoring tool for therapeutic effects [22]. The indication of surgical correction of leg deformities is based on radiographic assessment (Fig. 5a).

### Achondroplasia

**Fig. 6:**
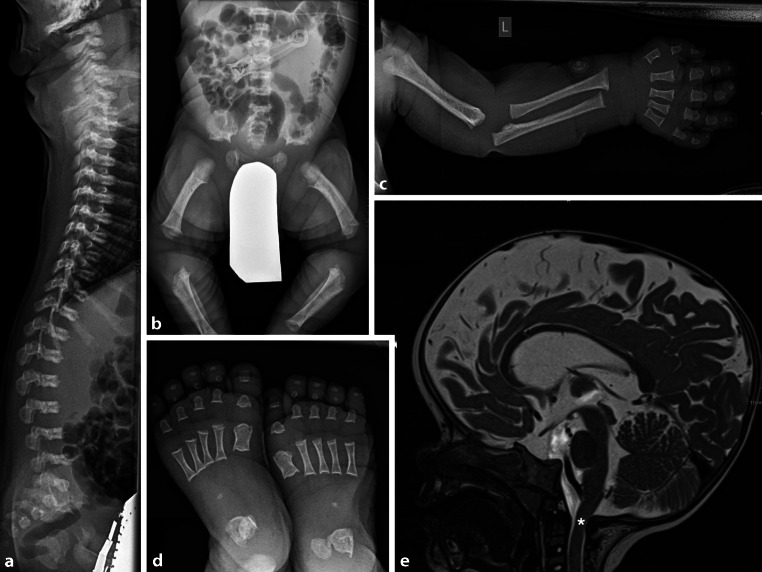
Achondroplasia.** a** Vertebral bodies with flattening, anterior beaking and thoracolumbar kyphosis. **b** Dysmorphic pelvis. **c** Rhizomelia and trident hand. **d** Dysmorphic metatarsals and phalanges. **e** MRI with foramen magnum stenosis (*asterisk*)

Similar to OI, achondroplasia is nowadays mostly diagnosed antenatally. Prenatal ultrasound and postnatal skeletal survey support the genetic diagnosis with the following features: frontal bossing, flat nasal bridge, rhizomelia, anterior vertebral beaking or wedging, dysmorphic pelvis with squared iliac wings and horizontal acetabular roofs (Fig. 6a–d). MRI of the craniocervical junction is needed to determine the extent of foramen magnum stenosis and cord compression (Fig. 6e). There is an ongoing discussion about the optimal timepoint of imaging, the clinical relevance of signal changes within the cord, and the standardization of follow-up intervals for MRI of the craniocervical junction [23, 24].

## New horizons for diagnostic imaging in children with bone and growth disorders—selected examples

Over the last few decades, biomedical imaging has been experiencing continuous improvements in methodology and applications. Here, selected methods of interest for the pediatric bone community are listed.

### Artificial intelligence

While there is large public and scientific interest in artificial intelligence (AI), only limited applications have made their way into pediatric imaging. One of the most successful examples of AI in clinical use is automated bone age assessment by a commercially available detection algorithm and software (“BoneXpert”, Visiana, Denmark) [25]. In the next decade, automated analyses of medical images by computer vision is expected to expand significantly and to continue its way towards clinical use.

### Assessment of bone microarchitecture by high-resolution peripheral quantitative computed tomography

High-resolution peripheral quantitative computed tomography (HR-pQCT) is a research imaging tool which provides quantitative measures of geometry, density, and bone microarchitecture of the peripheral skeleton. Specifically, the distal and ultradistal radius and tibia can be measured. Cortical bone and trabecular bone can be separately analyzed. The radiation dose is low (effective dose < 4 µSv) [26, 27]. Based on high nominal image resolution (isotropic voxel size 82 µm), advanced postprocessing techniques such as finite element analysis can be applied. Finite element analysis allows the simulation of falls and calculates biomechanical parameters of bone strength (e.g., stiffness; failure load).

**Fig. 7:**
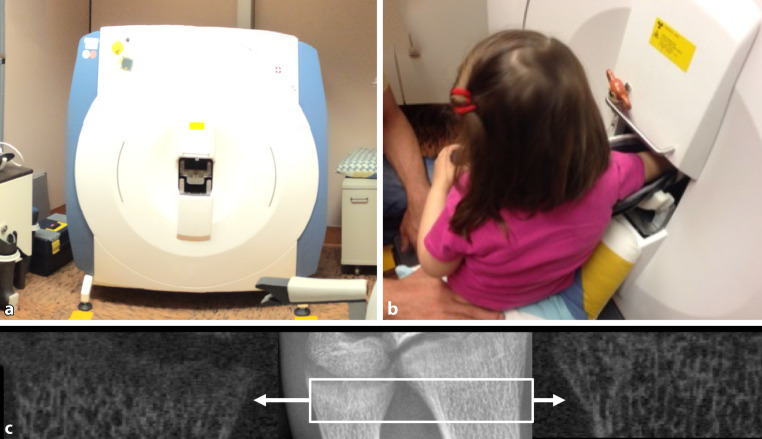
High-resolution peripheral quantitative computed tomography (HR-pQCT): **a** scanner, **b** pediatric positioning in generation I scanner. **c** Microarchitecture of the ultradistal ulna with abnormal physis (*left*) and bone microarchitecture of the ultradistal radius (*right*) in a child with XLH. *Center*: X‑ray of the same child. *Box* indicates HR-pQCT scan region shown on both sides

The majority of HR-pQCT research has been done in adults but a significant number of studies have now been published in children and adolescents. Although the radiation dose of HR-pQCT is low and scan time is fast, CT-based research remains complex in children. With longitudinal studies being complicated by growth, it is understandable that the definition of the most optimal region of interest remains a point of discussion among pediatric HR-pQCT users. While initial imaging protocols were similar to adult positioning (with fixed offsets relative to the joint surface) (Fig. 7), novel approaches integrate limb length and relative positioning (to either joint surface or the growth plate).

### Quantitative ultrasound in bone research

Quantitative bone ultrasound (QUS) is not a new method in bone research. Other than “conventional ultrasound” QUS provides numbers as opposed to images. In the last few decades, a significant number of studies supporting its value in the assessment of impaired bone strength have been published [28]. Traditional parameters derived from QUS include broadband ultrasound attenuation (BUA) and speed of sound (SOS). Evidence shows that scan sites depend on the device and software used. Numerous devices have been and are available on the market (using either axial or trough-transmission modes). Unfortunately, almost all of them are offered by different small vendors. More recently, the so-called bi-directional axial transmission (BDAT) technique has been developed and validated [29]. The velocity of the first-arriving signal measured by BDAT ultrasound of the distal extremities (radius and tibia) provides a surrogate for local cortical bone quality. It reflects cortical stiffness, cortical porosity (Ct.Po), and cortical thickness (Ct.Th) by a single integrative measure. We have recently used BDAT in children and adolescents with XLH and heathy controls. As hypothezised, there were significant differences in the velocity of the first-arriving signal between the groups [30]. The value of BDAT in follow-up and monitoring of XLH remains to be determined in the future.

## Conclusion

Advances in technical knowledge and imaging methods will facilitate improvements in diagnostic work-up of children with bone and growth disorders. However, pediatric radiologists with a musculoskeletal focus are needed for true interdisciplinary care of these patients.
